# Community-Based Ototoxicity Monitoring for Drug-Resistant Tuberculosis in South Africa: An Evaluation Study

**DOI:** 10.3390/ijerph182111342

**Published:** 2021-10-28

**Authors:** Lucia Jane Stevenson, Leigh Biagio-de Jager, Marien Alet Graham, De Wet Swanepoel

**Affiliations:** 1 Department of Speech-Language Pathology and Audiology, Faculty of Humanities, University of Pretoria, Corner of Lynnwood and University Roads, Hatfield, Pretoria 0002, South Africa; leigh.biagio@up.ac.za (L.B.-d.J.); dewet.swanepoel@up.ac.za (D.W.S.); 2 Department of Science, Mathematics and Technology Education, Faculty of Education, University of Pretoria, Corner of George Storrar Dr and Leyds St., Groenkloof, Pretoria 0002, South Africa; marien.graham@up.ac.za; 3 Ear Science Institute Australia, Perth, WA 6008, Australia

**Keywords:** community-based services, community health workers, decentralised services, drug-resistant tuberculosis, tuberculosis, hearing loss, ototoxicity monitoring, audiometry, South Africa

## Abstract

In response to the drug-resistant tuberculosis (DRTB) ototoxicity burden in South Africa, ototoxicity monitoring has been decentralised, with community health workers (CHWs) acting as facilitators. This study describes a community-based ototoxicity monitoring programme (OMP) for patients with DRTB. Findings are compared to the recommended guidelines for ototoxicity monitoring, the OMP protocol and published studies. This was a retrospective study of longitudinal ototoxicity monitoring of 831 patients with DRTB, using data collected at community-based clinics in the City of Cape Town between 2013 and 2017. Approximately half (46.8%) of the patients had an initial assessment conducted in accordance with the OMP protocol recommendations, and follow-up rates (79.5%) were higher than those of a similar DRTB programme. However, patients in this study were not monitored within the timeframes or with the regularity recommended by the guidelines or the OMP protocol. Extended high-frequency pure-tone audiometry (27.5%) was underutilised by testers and data recording was inconsistent (e.g., 37.7% of patient gender was not recorded by testers). Community-based OMP using CHWs to facilitate monitoring showed improvement over previous hospital-based reports, with more accessible services and higher follow-up rates. However, to improve OMP outcomes, OMP managers should reassess current protocols and data recording practices.

## 1. Introduction

An estimated 10 million people globally fell ill with tuberculosis (TB) in 2019 and South Africa has been identified as one of the eight countries that make up two-thirds of these cases [1]. With an estimated 615 people per 100,000 presenting with TB, compared to the global estimate of 130 cases per 100,000 [1], South Africa is recognised as one of 30 countries with a high burden of TB [1]. Despite advances in the effective diagnosis and treatment of TB, it is the leading cause of death in the country [2]. South Africa is furthermore afflicted by a high incidence of TB/human immunodeficiency virus (HIV) coinfection, with more than half (58%) of new and relapsed patients with TB being reported as HIV positive in 2019 [1,3].

Tuberculosis that is resistant to first-line anti-TB drugs is known as drug-resistant TB (DRTB), the main types of which are multi-drug-resistant TB (MDRTB), extensive drug-resistant TB (XDRTB) and Rifampicin-resistant TB (RRTB) [4,5]. In 2019, half a million people globally developed MDRTB/RRTB, with an estimated 14,000 cases in South Africa [1]. The emergence of drug-resistant strains has complicated TB control; never before have more people globally been affected by MDRTB [6], with numbers set to rise in high-burden countries in the coming decades [7,8].

Treatment of DRTB is complex and challenging for the patient, health care providers and for the health system [6]. Historically, patients with DRTB have required prolonged treatment, lasting two years or more [8], with the use of toxic second-line aminoglycosides, including Kanamycin [9,10]. Aminoglycosides are often used in developing countries for the treatment of DRTB because they have advantages over other classes of antibiotics and are inexpensive to produce [11,12]. Aminoglycosides are known to be toxic to both the vestibular and cochlear structures of the ear and to divisions of the eighth cranial nerve and the connections within the central nervous system [13,14]. The effects of cochleotoxicity result in permanent hearing loss [15,16] and/or tinnitus caused by the death of cochlear outer hair cells [9,13,17]. Encouragingly, injectable-free, non-aminoglycoside treatment regimens comprising shorter treatment durations with novel and repurposed drugs such as Bedaquiline, are being routinely phased in in South Africa for patients meeting specific eligibility criteria [4,18]. However, more than half the high-burden TB countries surveyed in 2020 were still using toxic injectable medicines, with 46% of countries reporting the use of Kanamycin and/or Capreomycin in the treatment of DRTB, counter to the most recent World Health Organisation (WHO, Geneva, Switzerland) recommendations [19]. As a result, a significant portion of the TB population may still be affected by aminoglycoside-induced hearing loss [16].

Ototoxic hearing loss has a negative impact on an individual’s social participation, emotional and behavioural well-being, quality of life, activities of daily living and employment status [9,14]. As a result, the monitoring of hearing during DRTB treatment is recommended [20,21,22] to ensure that hearing loss is detected early and that appropriate medical and rehabilitative intervention is implemented to mitigate the potential loss and negative effects [23,24]. Through the implementation of an audiological ototoxicity monitoring programme (OMP), ototoxicity is determined by comparing pure tone hearing thresholds, ideally obtained prior to the initiation of treatment and known as a baseline assessment, to subsequent hearing threshold monitoring measurements [15]. Weekly to monthly [20,21,22] monitoring is recommended following the baseline evaluation. A change in hearing thresholds that meets predetermined criteria for the presence of an ototoxic shift may offer medical personnel the opportunity to alter the treatment regimen [6,25]. Management strategies can be implemented as soon as a hearing loss is identified to eliminate its negative consequences and to ensure improved outcomes for patients [6]. Avoiding or minimising ototoxic hearing loss in patients with DRTB requires a combined approach of serial audiological monitoring and a tailored treatment regimen, which remains a significant challenge globally [25].

The significant burden of DRTB in South Africa has necessitated careful consideration of appropriate strategies for effective ototoxicity monitoring. Strategies that have been implemented include, amongst others, the introduction of new drugs and treatment regimens, the decentralisation of TB and ototoxicity monitoring services, the inclusion of community health workers (CHWs) in the model of care and the supply of audiological ototoxicity monitoring equipment and training. A decentralised model of care has been included as part of national policy for DRTB management to complement the capacity of centralised TB hospitals, to increase access to care and to improve treatment outcomes for patients with DRTB [10,26,27]. Decentralisation of services allows patients to access DRTB treatment on an outpatient basis at a facility nearest to them, such as a primary healthcare (PHC) or community health clinic, instead of at a centralised TB hospital. Outpatient units have been tasked with initiating and administering treatment, including offering audiological ototoxicity monitoring [10], and monitoring any adverse side effects of the treatment.

With advances in portable audiometric technology, hearing assessments can be conducted at PHC and community level with limited training and resources [28]. In response to the high incidence of ototoxic hearing loss in patients with DRTB, South Africa implemented a national ototoxicity prevention programme to improve access to audiological monitoring and to reduce ototoxic hearing loss [28]. As part of this programme, training and portable automated audiometers were provided to selected health facilities, including PHC and community health clinics. This has reduced the waiting time for patients wanting to be assessed and linked to rehabilitative audiological services [28]. In order to address the shortage and poor distribution of healthcare workers in resource-constrained settings, the employment of CHWs has been proposed [29]. The South African department of health has recognised the need to expand the PHC system by integrating 50,000 CHWs into the public health system between 2019 and 2024 to improve access to services; [30]. CHW include a variety of community health aides who are trained and working in their own communities [31]. CHWs engage in task-sharing that involves the appropriate reallocation of tasks to nonspecialists, such as hearing assessments that are traditionally performed by ear and hearing specialists [28]. The use of CHWs, supported by innovative technologies has demonstrated improved access to ear and hearing care services [15,32,33] and, together with a public health approach can offer a solution to the limited human resources available. [28]

Despite South African DRTB treatment guidelines recommending that all patients receiving treatment should undergo ototoxicity monitoring [10], historically a lack of OMPs has seen very few patients being monitored [34,35]. The development of OMPs in South Africa has been hindered by a number of obstacles including a lack of human and material resources necessary for ototoxicity monitoring [34,35,36,37,38], poor collaboration amongst healthcare professionals treating patients, and patient-related barriers such as a lack of awareness of treatment side effects and difficulties travelling to ototoxicity monitoring service locations [39]. Up until 2018, when the official South African guidelines for ototoxicity monitoring were introduced [22], international guidelines [20,21] were modified by health care providers to suit the South African context, leading to considerable variation in their application [37]. Recent reports indicate, however, that where OMPs do exist, the assessment and management practices of audiologists of patients on ototoxic medication do not align with guideline recommendations [40] and that outpatient-based ototoxicity monitoring services are underused by patients [41]. The COVID-19 pandemic has exacerbated the challenges of treating TB and the monitoring of associated ototoxicity because of the extra burden on health care services, care seeking behaviour and the reallocation of human, financial and other resources from TB to COVID-19 care [1].

Monitoring the effectiveness of ototoxicity monitoring services and reporting on the practices of existing OMPs is essential to support evidenced-based health care [22] and to optimise and improve care [35]. This study aimed to describe the service delivery practices of a decentralised, community-based OMP for DRTB, including a comparison between CHWs and PHC audiologists facilitating the ototoxicity monitoring. The practices of this real-world community-based OMP were compared to the national and international guidelines for ototoxicity monitoring and to the OMP protocol to identify successes and pitfalls with the aim of improving services and guiding future OMP implementations. To our knowledge, this is the first study to report on ototoxicity monitoring for DRTB conducted by CHWs in a decentralised community-based model of care for increased patient access.

## 2. Materials and Methods

This was a longitudinal retrospective study of ototoxicity monitoring of patients with DRTB between 2013 and 2017. The study aimed to describe the practices of community-based OMP for patients with DRTB, focusing on the following aspects: the timing and frequency of ototoxicity monitoring assessments, the follow-up rates of the program, the ototoxicity monitoring assessment methods used and the OMP data management procedures. The findings of the OMP practices were compared to the most widely used recommended guidelines for ototoxicity monitoring and management [20,21,22], to the OMP protocol and to other comparable published studies. Data collected by CHWs were compared to data collected by audiologists in PHC.

### 2.1. Participants

This study used data collected at outpatient community-based clinics in two subdistricts of the City of Cape Town, namely the Mitchells Plain/Klipfontein and the Western/Southern subdistricts. In 2012, a pilot project to upgrade the skills of 30 existing CHWs in the field of rehabilitation was implemented in the Western Cape in order to improve PHC and community-based rehabilitation for people with disabilities [42]. This new category of CHWs was trained to conduct ototoxicity monitoring, amongst other tasks, and is known as rehabilitation care workers. The Mitchells Plain/Klipfontein and the Western/Southern subdistricts were selected for inclusion in this study because both the upskilled CHW and PHC audiologists were the active testers in these areas. They used conventional pure-tone audiometry and/or extended high-frequency pure-tone audiometry for ototoxicity monitoring associated with DRTB. Nonprobability purposive sampling was used to select all patients with DRTB, regardless of age or gender, who were enrolled in the OMP between May 2013 and September 2017. The patient interviews and ototoxicity monitoring assessments were conducted by testers at 19 PHC and community health clinics in the two subdistricts.

### 2.2. Procedures

**Figure 1:**
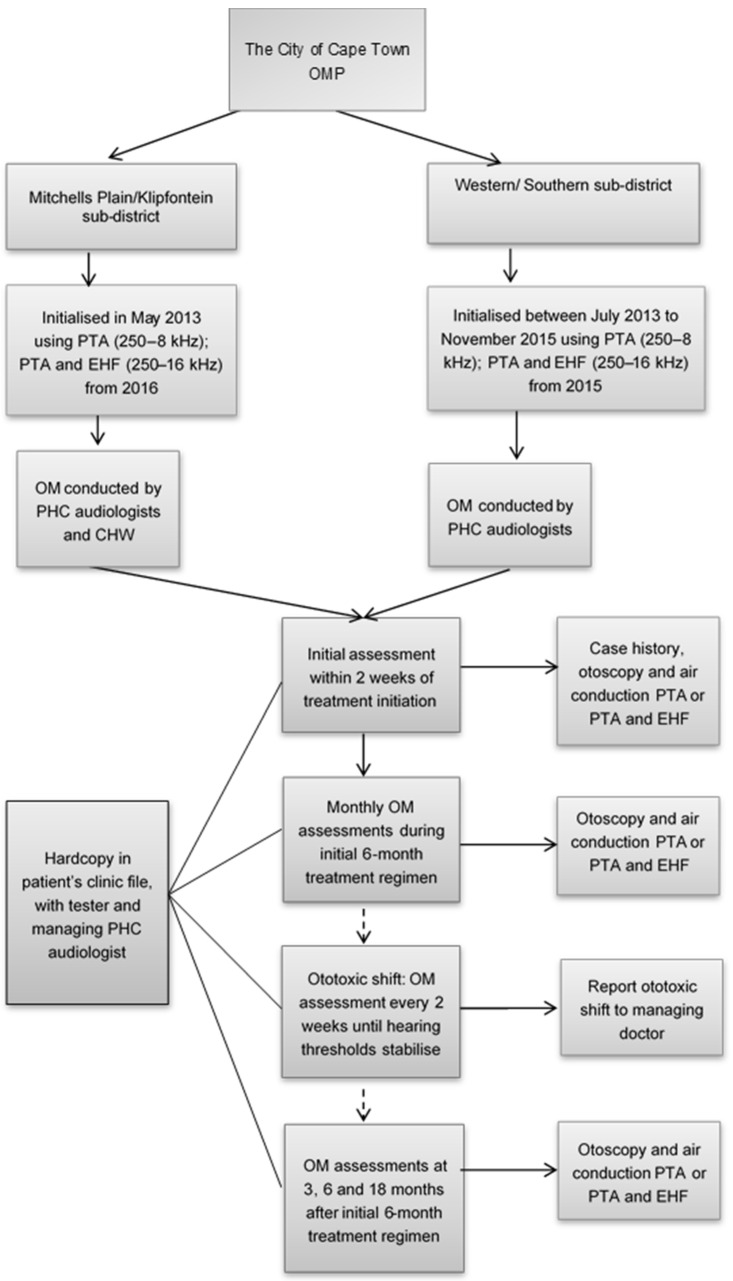
The City of Cape Town OMP protocol. OMP, ototoxicity monitoring programme; PTA, pure-tone audiometry; EHF, extended high-frequency pure-tone audiometry; OM, ototoxicity monitoring; PHC, primary healthcare; CHW, community health worker.

The OMP protocol that was implemented at the time of data collection is outlined in Figure 1. All patients who received ototoxic medication for treatment of DRTB were identified and referred by their managing doctor and included in the OMP as part of the package of care. Patients visited a PHC or community health clinic daily for the first six months of treatment to receive their medication from a nurse. After the initial six-month treatment period, medication was continued for 18 months with patients visiting a clinic weekly to obtain their medication, and monthly to consult with their managing doctor.

Testers travelled to the clinics in each subdistrict with portable audiological equipment. The KUDUwave audiometer (eMoyo, Johannesburg, South Africa) was used by testers in this study. It is a portable, PC (Dell laptop, Dell Inc., Round Rock, TX, USA)-controlled clinical diagnostic audiometer and integrated supra-aural ear cup and insert earphone headset and electronic response button for use without a soundproof booth. Automated and manual programs conduct audiometry up to 16,000 Hz. Results are stored electronically and store-and-forward for printing. PHC audiologists and CHW were testers in the Michell’s Plain/Klipfontein subdistrict whereas only PHC audiologists were testers in the western/southern subdistrict. At the time of a patient’s initial assessment, identifying data including the patient’s name, date of birth, gender and medical history pertaining to HIV status, DRTB medication/s, comorbidities and adverse effects were recorded manually on a paper data collection form by the tester. This information was obtained from the patient’s medical records in a clinic file and/or verbally reported to the tester during the patient interview. A bilateral otoscopic examination was conducted and the findings recorded on the data collection form. If pathology was suspected, the patient was referred to the managing doctor or nurse for appropriate treatment, in addition to audiometry, as per the OMP protocol.

At the time of data collection, the official South African ototoxicity monitoring guidelines had not yet been published, thus health care providers relied on adaptations of the international guidelines of the American Speech-Language-Hearing Association (ASHA, Rockville, MD, USA) [20] and the American Academy of Audiology (AAA, Reston, VA, USA) [21] when developing the OMP procedure protocol. An unpublished draft of the Health Professions Council of South Africa’s (HPCSA) ototoxicity monitoring guidelines [22] was, however, available to the OMP developers to assist them in applying the international guidelines to the South African context.

The protocol followed by the OMP at the time of data collection is outlined in Figure 1. Initial assessments were conducted at the clinics within two weeks of the DRTB treatment initiation. Monitoring assessments were conducted once a month during the initial six-month treatment regimen and then at three, six and 18 months thereafter. The timing of the initial and monitoring assessments was determined by the OMP managers to best suit the community-based nature of the OMP where testers had to travel to numerous clinics on a rotational basis. Where an ototoxic shift meeting predetermined criteria [20] was evident, the managing doctor was informed immediately and monitoring assessments were then conducted every two weeks until no change in hearing thresholds was detected. Assessments were conducted in a quiet environment and included bilateral pure-tone audiometry (250–8 kHz), or pure-tone audiometry and extended high-frequency pure-tone audiometry (250–16 kHz) if available. The equipment required to conduct both pure-tone audiometry and extended high-frequency pure-tone audiometry became available in November 2015 at the southern/western subdistrict and in July 2016 at the Mitchell’s Plain/Klipfontein subdistrict; prior to this, only pure-tone audiometry was available for ototoxicity monitoring. Typically, manual testing would have been done; however, an automatic mode of threshold determination may also have been used in some instances.

Each patient’s descriptive and audiological data were recorded manually by the testers on paper-based data collection forms and stored in the patient’s clinic file. A copy of each patient’s data collection form was kept with the tester and regularly made available to the managing PHC audiologist responsible for each subdistrict for review. Upon completion of a patient’s DRTB treatment and ototoxicity monitoring, the data collection form was stored permanently with the PHC audiologist responsible for each subdistrict. The researchers collected the hard copies of the patients’ data collection forms from the managing PHC audiologists in each subdistrict for analysis and these were returned upon completion of this study.

### 2.3. Data Analysis

Data were imported from Excel into Statistical Package for Social Sciences (SPSS, IBM Corp. Armonk, NY, USA) software (version 27), after which descriptive statistics such as frequency distributions, weighted arithmetic mean, measures of central tendency, variability and relationships (correlations) were used to present and interpret the data in a meaningful way. Frequencies and cross-tabulations were compiled to describe the patient sample. The two proportions z-test was used to determine whether two proportions of two groups (patients who were assigned a follow up return date and those who were not) differed significantly on one characteristic, the follow up return rate. A multivariate logistic regression model was built, with the dependent variable being dichotomous (whether a patient would follow-up after the initial test or not). The Nagelkerke R^2^ was used to determine the percentage of variation of the dependent variable which was explained by the predictors (age, gender, treatment duration and HIV status).

The OMP used paper-based data collection forms that were manually completed by the tester for each patient. However, the collection of data by testers describing the patients and their treatment regimens was sporadic. Where important data were missing, this was because it was not recorded on the data collection forms by the testers and was therefore unavailable to the researchers for inclusion in this retrospective study. Many of the patients (37.7%; *n* = 313) did not have their gender recorded on their data collection forms and for almost a fifth of patients (18.7%; *n* = 155) no DRTB medication type and/or date indicating when their treatment was initiated was recorded (27.6%; *n* = 229). Thus, treatment duration could only be determined for a minority of patients (14.2%; *n* = 118) for whom both treatment initiation and end dates had been recorded on their data collection forms.

## 3. Results

### 3.1. Participants

A total of 831 DRTB patients who attended ototoxicity monitoring services between 2013 and 2017 was included as patients in this study. The patients’ ages (798/831 (this format denotes *n*/group total)) ranged from 12.3 to 68 years with a mean of 36.1 years (standard deviation (SD) = 11.00). CHWs assessed 60.3% of patients (501/831), whereas the remaining 39.7% patients (330/831) were assessed by PHC audiologists. Of the 676 patients whose medication had been recorded, 99.4% (672/676) were administered Kanamycin. Only 2.2% of patients (15/676) had more than one medication recorded, therefore only the primary medication administered was used to determine the duration of treatment and to report on the timing of ototoxicity monitoring assessments in relation to treatment initiation. At the time of the initial assessment, 29.1% of patients (242/831) reported having TB/HIV coinfection and 24.1% (200/831) had the use of antiretroviral medication recorded on their data collection forms (Table 1). Where treatment initiation and end dates were recorded, treatment duration ranged from six to 596 days with a mean of 160.5 days (SD = 106.84).

### 3.2. Timing and Frequency of Ototoxicity Assessments

A total of 72.4% of the patients (602/831) had a treatment initiation date recorded by the tester on their data collection form (Table 1). Almost half (46.8%; 282/602) of the patients had had an initial assessment conducted prior to or within two weeks of treatment initiation, in accordance with the OMP protocol recommendation (Table 2); 89.9% of patients (541/602) had an initial assessment conducted after starting their treatment and had been receiving their medication for more than two months (70.3 days; SD = 131.50) before undergoing an initial assessment (Table 2).

**Figure 2:**
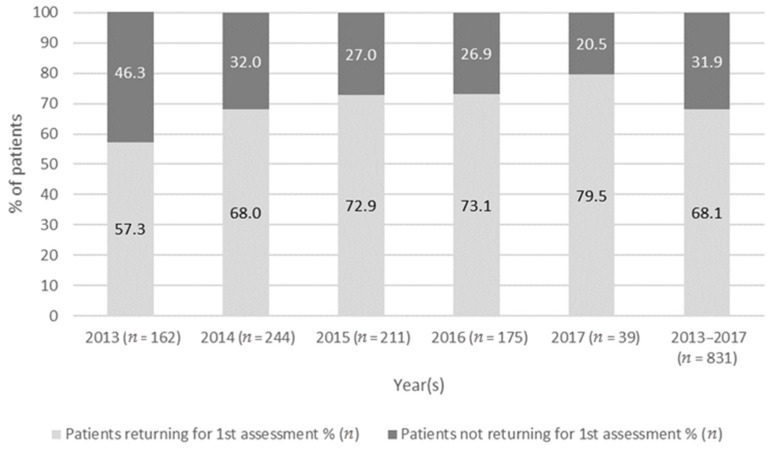
Follow-up rates of the first monitoring assessment between years 2013–2017 (*n* = 831).

**Figure 3:**
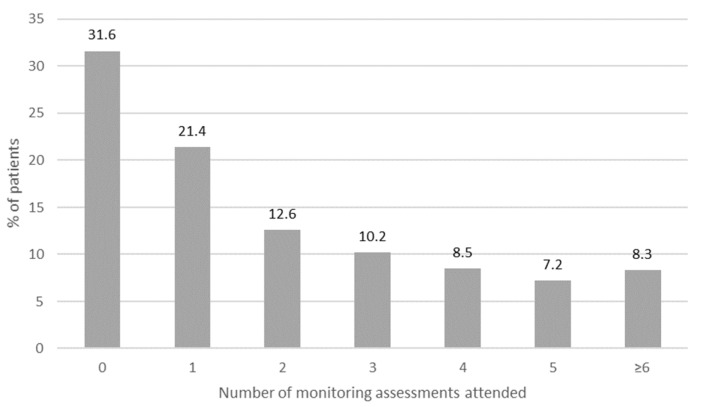
Distribution of the number of monitoring assessments attended by patients (*n* = 831).

Follow-up default rates ranged from 27.6 to 31.9% across consecutive monitoring assessments (Table 3). Follow-up rates improved from 53.7 to 79.5% from 2013 to 2017 (Figure 2). On average, patients were assessed 3.1 (SD = 2.31) times but 31.6% (263/831) attended an initial assessment only and just 8% (69/831) returned for the recommended [20,21,22] six or more ototoxicity monitoring assessments (Figure 3).

Multivariate logistic regression models showed that gender, age, HIV status, and the duration of administration of medication did not have a significant effect on follow-up rates (*p* > 0.05). Patients who were given a specific date (25.0%; 208/831) on which to return for an ototoxicity monitoring assessment did not have a significantly better follow-up return rate (two-proportions z-test; *p* = 0.052) either. Once extended high-frequency pure-tone audiometry was introduced to the OMP in 2015, 27.5% of patients (117/425) making OMP visits had their hearing assessed using extended high-frequency pure-tone audiometry as well as pure-tone audiometry.

A comparison of patients assessed by CHWs and PHC audiologists is presented in Table 4. The timing and frequency of ototoxicity monitoring was similar for the two groups of testers. PHC audiologists were more likely to use both pure-tone audiometry and extended high-frequency pure-tone audiometry for the initial assessment of patients, however. The findings of this study were compared to the OMP protocol and national and international guideline recommendations, as reflected in Table 5.

## 4. Discussion

Almost half (46.8%) of DRTB patients had an initial assessment conducted in accordance with the OMP protocol recommendation, before or within 14 days of treatment initiation. This is more positive compared to a previous South African hospital-based study that reported that only 10% of patients could be tested within two weeks of treatment initiation [35]. The follow-up rates for the first three assessments ranged from 68.1 to 72.4%. Encouragingly, the follow-up rates between the initial assessment and first monitoring assessment improved to 79.5% as the OMP became more established from 2013 to 2017. The follow-up rates of this study are higher than those of a community-based DRTB treatment program that included ototoxicity monitoring, where the loss to follow-up was reported as being as high as 38% [43]. This demonstrates the potential of a community-based model of care for ototoxicity monitoring to establish itself over time as a robust, widely used service. Similar timing and frequency of ototoxicity monitoring was found in patients assessed by CHW and those assessed by PHC audiologists. Therefore, the findings of the current study support the use of CHW to facilitate community-based ototoxicity monitoring of patients with DRTB.

Despite improvements in ototoxicity monitoring service delivery using community-based care and CHWs to facilitate monitoring, the OMP still falls short in several areas. The findings indicate that the OMP was unable to meet the outcomes set out by the guidelines [20,21,22] and OMP protocol, supporting existing reports [41]. One of the indicators of quality and effectiveness of an OMP is the timely assessment and monitoring of patients who may develop ototoxic hearing loss [21,22]. The timing of initial assessments in the current study did not meet the guideline or OMP protocol recommendations for more than half (53.2%) of the patients. Most patients (89.9%) received their medication more than two months (Average = 70.3 days; SD = 131.50) before undergoing an initial assessment. This far exceeds the guideline and OMP recommendations [20,21,22], which state that an initial assessment should be conducted prior to, or within three to 14 days of treatment initiation. Timely initial assessments are vital to effective ototoxicity monitoring. Subsequent monitoring measures are compared to those obtained during the initial assessment and any decisions regarding counselling, the adjustment of treatment regimens or substitution with less ototoxic drugs are based on these comparisons [44]. Historically, the recording of timeous initial assessments has been inconsistent, as reported by South African OMPs [35,37,40,45] and evidenced in the current study.

Patients in this study were not monitored with the regularity recommended by the guidelines and the OMP protocol. More than 90% did not attend the recommended six or more monthly monitoring assessments [20,21,22]. Throughout the course of DRTB treatment, lasting up to 18 months and in some cases even longer, patients were assessed on average only 3.07 (SD = 2.31) times. Ototoxicity monitoring was conducted on average every 58.3 (SD = 6.23) days, almost twice the 30 days recommended by the OMP protocol. This undermines the purpose of ototoxicity monitoring and results in a missed opportunity for early detection and management of ototoxic hearing loss. Previous reports have also indicated that audiologists conducting ototoxicity monitoring in South Africa do not conduct monitoring assessments with the frequency recommended by the national guidelines, that is, every two weeks [40]. These poorly met indicators raise questions about the effectiveness of OMPs and suggest that careful review and reconsideration of approaches, technologies and human resources used is required.

When extended high-frequency pure-tone audiometry was made available to the testers in this study it was underutilised, with less than a third of patients (27.5%) assessed undergoing using extended high-frequency pure-tone audiometry. Extended high-frequency pure-tone audiometry has been recommended for ototoxicity monitoring as a method to detect hearing damage earlier than conventional pure-tone audiometry [20,21,22]. Historically, most audiologists in South Africa have not used extended high-frequency pure-tone audiometry when conducting ototoxicity monitoring because the specialised equipment was unavailable to them [37]. Even when available, extended high-frequency pure-tone audiometry is often used inconsistently throughout a patient’s course of treatment, making reliable comparisons of hearing thresholds difficult or impossible [40].

It is unclear why testers in this study did not use the extended high-frequency pure-tone audiometry for ototoxicity monitoring when it was available. One reason may relate to the additional time required and the difficulty in performing consecutive tests for chronically ill patients [36]. It is vital to conduct a quick and efficient hearing assessment on patients with DRTB as reliable behavioural responses are needed to make accurate comparisons for ototoxicity detection [36]. Assessment procedures may need to be adapted for patients unable to cope with a comprehensive assessment [22]. A sensible recommendation [21,22] has been to implement individualised, shortened, serial monitoring protocols that target the highest frequencies most sensitive to ototoxicity [46], or to reduce the number of frequencies assessed to include the high frequencies only [22]. The use of a sensitive range for ototoxicity has been shown to decrease test time to one third that of a comprehensive test of all frequencies [46]. The use objective, noninvasive distortion product otoacoustic emission testing could be considered as an ototoxicity monitoring assessment tool, particularly for difficult-to-test patients [36]. Distortion product otoacoustic emission testing offers a quick, reliable, cost-effective method to detect initial cochlear ototoxic changes before they are able to be detected by conventional pure-tone audiometry [36]. The application of such protocols and adaptions could alleviate the strain on time and human resources synonymous with ototoxicity monitoring [46], leading to more successful OMP outcomes. In addition, the use of an automated test protocol together with a smartphone based mobile technology may further support time effective assessments [33]. These findings highlight the importance of ongoing quality control measures and supportive supervision strategies [29] for OMP as well as the continuous training of testers [47], including task-shifting, to facilitate assessments.

In this study, the data recorded by testers on paper-based data collection forms were inconsistent. The descriptive data for more than a third (37.7%) of the patients were unavailable to the researchers for retrospective analysis. In South Africa, where a systematic national electronic health data management system for ototoxicity monitoring does not exist, it is common practice for audiologists conducting ototoxicity monitoring services to rely solely on paper-based data management procedures [39]. This can lead to errors in collecting and analysing data [39], as evidenced in this study. Thorough data collection and management in the field are necessary for auditing and research purposes, and are of particular importance when comparing repeated measures such as those of an OMP [39]. The ongoing training and monitoring of testers is important to maintaining a high standard of data collection and management for OMPs [47]. The use of smartphone technology and cloud-based data management has been shown to offer effective data management for large scale screening purposes [47]. Integrating secure data sharing with national health repositories should be considered to improve data management procedures of OMPs in South Africa [33].

Limitations of this study included a high rate of data that were unavailable for analysis and the lack of quantitative measures of the quality of testing by CHWs and PHC audiologists. Furthermore, patient interviews were conducted at the initial assessment only, and not at subsequent monitoring and exit assessments; after prolonged treatment with Kanamycin, the incidence of self-reported adverse audiological symptoms may have been higher.

## 5. Conclusions

Community-based OMP using CHWs to facilitate monitoring showed improvement over previous hospital-based reports with higher follow-up rates and more accessible services. CHWs may also support OMP services by alleviating the strain on hospital-based services, particularly during the COVID-19 pandemic. However, to improve OMP outcomes and to encourage timely ototoxicity assessment, current protocols may require reassessment to optimise limited resources. The poor utilisation of extended pure-tone audiometry by testers suggests that a more targeted approach to ototoxicity monitoring is required, where only frequencies most sensitive to ototoxicity are prioritised. Mobile smartphone audiometry solutions with paperless cloud-based data management may further support decentralised monitoring facilitated by CHWs.

## Figures and Tables

**Table 1:** Patient description at the time of the initial assessment (*n* = 831).

Variable	%	*n*/Group Total
Gender		
Not recorded	37.7	313/831
Male	57.3	297/518
Female	42.7	221/518
Treatment regimen		
Not recorded	18.7	155/831
Kanamycin	99.4	672/676
Capreomycin, Azithromycin or Amikacin	0.6	4/676
More than one medication	2.2	15/676
Treatment duration		
Treatment initiation date not recorded	27.6	229/831
Treatment initiation date recorded	72.4	602/831
Risk factor for ototoxicity		
TB/HIV coinfection	29.1	242/831
Antiretroviral treatment	24.1	200/831
Noise exposure	14.9	124/831
Audiological symptoms		
Tinnitus	18.2	151/831
Hearing loss	10.2	85/831
Aural fullness	8.5	71/831
Wax impaction		
Left ear	11.1	92/831
Right ear	11.7	97/831
Tester		
PHC audiologist	39.7	330/831
CHW	60.3	501/831

TB/HIV, tuberculosis/human immunodeficiency virus; PHC, primary healthcare; CHW, community health worker.

**Table 2:** Timing of initial assessment in relation to treatment initiation (*n* = 602).

Timing of Initial Assessment	Prior to Treatment Initiation		Post Treatment Initiation			
	≥4 Weeks Prior to Treatment Initiation	Same Day as Treatment Initiation	1–3 Days Post Treatment Initiation	4–14 Days Post Treatment Initiation	2–4 Weeks Post Treatment Initiation	≥4 Weeks Post Treatment Initiation
Patients % (*n*)	4.5 (27)	5.6 (34)	9.0 (54)	27.7 (167)	18.6 (112)	34.6 (208)
Days from treatment initiation and initial assessment Average (SD)	163.6 (166.41)	0 (0)	70.3 (131.50)			

SD, standard deviation.

**Table 3:** Follow-up rates and days elapsing between the first three monitoring assessments.

Assessments	Initial to 1st Monitoring Assessment	1st to 2nd Monitoring Assessment	2nd to 3rd Monitoring Assessment
Follow-up rate % (n)	68.1 (566/831)	68.4 (387/566)	72.4 (280/387)
Average number of days between assessments Average (SD)	58.8 (79.03)	56.1 (81.23)	53.5 (62.55)

**Table 4:** Comparison of the audiometric protocol used, the timing and frequency of ototoxicity monitoring of patients assessed by CHWs and by PHC audiologists.

Variable	CHWs	PHC Audiologists
Patients assessed % (*n*)	60.3 (501/831)	39.7 (330/831)
Initial assessment conducted before, or 1–14 days of treatment initiation % (*n*)	45.6 (209/458)	50.7 (73/144)
Audiometric protocol for initial assessments % (*n*)	Years 2013–2017: PTA: 95.8 (480/501) Years 2015–2017: PTA and EHF: 8.3 (21/252)	Years 2013–2017: PTA: 70.9 (234/330) Years 2015–2017: PTA and EHF: 55.5 (96/173)
Follow-up rate for 1st monitoring assessment % (*n*)	68.5 (343/501)	67.6 (223/330)
Days between monitoring assessments Average (SD)	56.6 (5.62)	60.7 (9.45)
Average number of times a patient was assessed (SD)	3.0 (2.20)	3.1 (2.46)
Patients attending ≥6 monitoring assessments (%)	7.6	9.3

CHWs, community health workers; PHC, primary healthcare; PTA, pure-tone audiometry; EHF, extended high-frequency pure-tone audiometry.

**Table 5:** Current findings compared to the OMP protocol and guideline recommendations [20,21,22].

Principle	OMP/Guideline Recommendation				Current Findings % (*n*)
	ASHA	AAA	HPCSA	OMP	
Timing of initial assessment in relation to treatment initiation	Before treatment initiation or within 3 days of initiation	Before treatment initiation or within 3 days of initiation (Kanamycin)	Before treatment initiation or within 3 days of initiation	Before treatment initiation or 1–14 days after initiation	Before or within 3 days of treatment initiation: 19.1 (115/602) Before or 1–14 days after treatment initiation: 46.8 (282/602) ≥15 days after treatment initiation: 53.2 (320/602)
Audiometric protocol for initial assessments	PTA and EHF	PTA and EHF	PTA and EHF	Years 2015–2017: PTA and EHF	Years 2013–2017: PTA: 85.9 (714/831) Years 2015–2017: PTA and EHF: 27.5 (117/425)
Frequency of monitoring assessments during 2-year treatment period	Weekly, if possible, then monthly after treatment stops until hearing stabilises then at 3 and 6 months.	Weekly or biweekly. Cessation of monitoring is unspecified.	Biweekly then monthly after treatment ends until hearing stabilises then at 3 and 6 months.	Monthly for initial 6 months then at 3, 6 and 18 months post initial 6-month treatment period. This equates to at least 9 assessments.	Patients were assessed 3.1 times on average
Timing between monitoring assessments	7 days	7–14 days	14 days	30 days	58.3 (SD = 6.23) days on average
Monitoring assessment follow-up return rate	Not specified	Not specified	Not specified	Not specified	68.1–72.4% for the 1st–3rd monitoring assessments

OMP, ototoxicity monitoring programme; ASHA, American Speech-Language-Hearing Association; AAA, American Academy of Audiologists; HPCSA; Health Professions Council of South Africa; PTA, pure-tone audiometry; EHF, extended high-frequency pure-tone audiometry; SD, standard deviation.

## Data Availability

Data supporting reported results are stored at the University of Pretoria’s Department of Speech-language Pathology and Audiology and are available on request from the corresponding author. The data are not available publicly for ethical reasons pertaining to privacy, anonymity and confidentiality of the patients.
